# Surgical outcomes of laparoscopic total extraperitoneal (TEP) inguinal hernia repair compared with Lichtenstein tension-free open mesh inguinal hernia repair: A prospective randomized study

**DOI:** 10.1097/MD.0000000000029746

**Published:** 2022-06-30

**Authors:** Mohammed Yunus Shah, Pratik Raut, T.R.V. Wilkinson, Vijay Agrawal

**Affiliations:** a Department of Minimal Access, Bariatric and General Surgery, Al Ahli Hospital, Qatar University, Doha, Qatar; b Maharashtra University of Health Sciences, Maharashtra, India; c Department of Surgery, NKP Salve Medical College and Research Centre, Nagpur, Maharashtra, India.

**Keywords:** inguinal hernia, laparoscopy, laparoscopic TEP, Lichtenstein, mesh repair

## Abstract

Inguinal hernia repair is one of the most frequently performed surgery. The ideal procedure for inguinal hernia repair remains controversial. Open Lichtenstein tension-free mesh repair (LMR) is one of the most preferred open techniques with satisfactory outcomes. Laparoscopic approach in inguinal hernia surgery remains controversial, especially in comparison with open procedures. In this study, we have reported a comparison of laparoscopic total extraperitoneal (TEP) inguinal hernia repair with LMR. Postoperative pain, operative time, complications like seroma, wound infection, chronic groin pain, and recurrence rate were parameters to evaluate the outcome.

One hundred seventy-four patients were included in the study by consecutive randomized prospective sampling. The patients were divided into 2 groups: group A, laparoscopic TEP inguinal hernia repair, and group B, LMR. The procedures were performed by experienced surgeons. The primary outcomes were evaluated based on postoperative pain and recurrence rate. Secondary outcomes considered for evaluation were operative time, complications like seroma, infection, and chronic groin pain.

Severe pain was reported in group A (7.9%) compared to group B (15.1%), which was statistically significant (*P* < .001). Moderate pain was reported more in group B (70.9%) compared to group A (29.5%) (*P* < .001). The mean operative time in group A was 84.6 ± 32.2, which was significantly higher than that in group B, 59.2 ± 14.8. There was no major complication in both groups. The chronic pain postoperatively was significantly in higher number of patients in group B vs group A (22.09% vs 3.4%). The postoperative hospital stay period was significantly lesser for group A vs for group B (2.68 ± 1.52 vs 3.86 ± 6.16). Time duration taken to resume normal activities was significantly lower in group A (13.6 ± 6.8) vs (19.8 ± 4.6) in group B (*P* < .001).

Although there is definite evidence of longer operative time and learning curve, laparoscopic TEP has added advantages like less postoperative pain, early resumption of normal activities, less chronic groin pain, and comparable recurrence rate compared to open Lichtenstein repair. Laparoscopic TEP can be performed with acceptable outcomes and less postoperative complications if performed by experienced hands.

## 1. Introduction

Inguinal hernia repair is the most common operation performed by general surgeons.^[1]^ The definitive treatment of inguinal hernia is surgery. Various techniques have been described for inguinal hernia repair in the literature over the decades. The use of mesh has shown a significant reduction in recurrence rates.^[2]^

Lichtenstein et al^[3]^ described the use of mesh in the operative technique for tension-free inguinal hernia repair with satisfactory outcomes, which popularized the use of polypropylene mesh among the general surgeons.

The open Lichtenstein mesh repair of inguinal hernia has become a standard for inguinal hernia repair due to ease of performance along with low recurrence rates.^[4]^

Since the introduction of laparoscopic techniques in inguinal hernia repair, there have been controversies regarding safety, outcome, and feasibility of procedure. Laparoscopic techniques have shown acceptable results in a number of literature.^[5]^

The various techniques for inguinal hernia repair are still controversial and there is no consensus. Our aim was to perform a comparative study of laparoscopic total extraperitoneal (TEP) inguinal hernia repair with open Lichtenstein tension-free mesh repair (LMR) to evaluate the postoperative outcomes.

## 2. Methods

**Figure 1.**
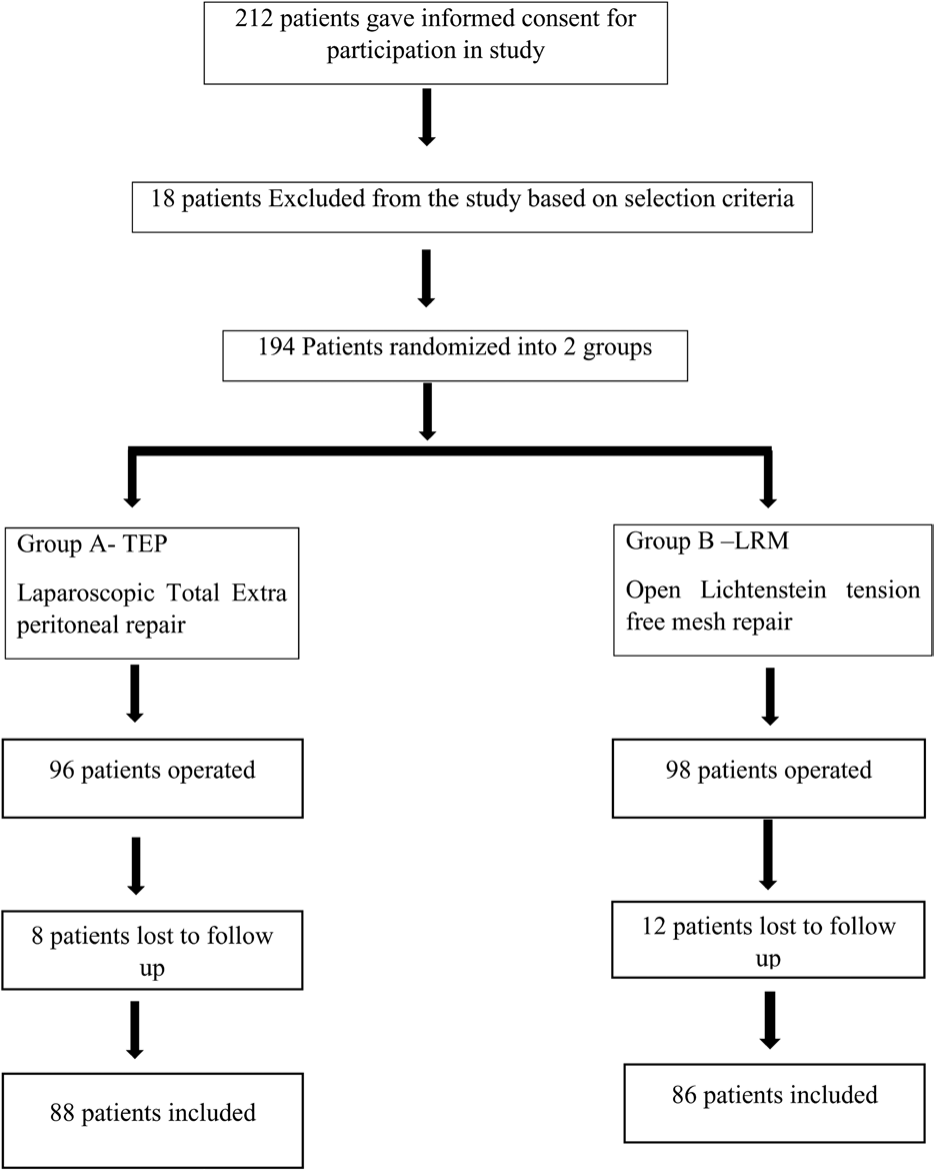
CONSORT chart showing the flow of participants through each stage of a randomized control study.

A randomized prospective study was conducted between June 2017 and June 2020 at NKP Salve Medical College and Research Centre and Lata Mangeshkar Hospital, Nagpur, Maharashtra, India. Two hundred twelve patients within the age group 18 to 80 years irrespective of gender, with unilateral inguinal hernia were included in the study after the ethical committee approval. Eighteen patients were excluded from the study based on the selection criteria like patients with recurrent inguinal hernias, obstructed hernias, strangulated, irreducible inguinal hernias, and bilateral inguinal hernias to maintain the homogeneity within the groups in respect to incisions and pain score evaluation. Also, patients not willing to give informed consent, not willing for follow-up, and unfit for anesthesia were also excluded from the study. Total 196 patients were operated in both the groups: 96 patients in the laparoscopic TEP group A and 98 patients in the LMR group B. During the postoperative follow-up period, 8 and 12 patients were lost to follow-up in group A and group B, respectively. So, a total of 174 patients were included in the study and alternatively randomized to laparoscopic TEP inguinal hernia repair (88 patients, TEP group A) and LMR (86 patients, LMR group B) as shown in Figure 1 consort chart showing the flow of participants through each stage of a randomized control study. All the confirmed eligible 174 patients were followed up on postoperative days 1, 7, 1 month, and 6 months, and outcomes measures were analyzed.

Both the procedures were performed by 2 experienced surgeons. The primary outcomes studied were postoperative pain and recurrence. Secondary outcomes measured were operative time, length of hospital stay, time to resume normal activities, and postoperative complications.

### 2.1. Surgical procedures

All the laparoscopic TEP procedures were performed under general anesthesia. A 10 mm blunt trocar was introduced after 1 cm infraumbilical incision and blunt separation of recti muscles were done to visualize the posterior rectus sheath. A 10-mm 0° telescope was introduced into the preperitoneal space and blunt dissection was done with the tip of the scope with CO_2_ pressure at 14 mm Hg, following the white loose areolar tissue plane. After sufficient preperitoneal space was created, the 0° telescope was replaced by 30° telescope.

Two 5-mm working ports were introduced in the midline. Blunt dissection of the preperitoneal space was done. After complete dissection along the Coopers ligament to the femoral canal, the hernia sac was dissected and reduced completely. In some cases of complete sac, it was transected and ligated with endoloop. The myopectineal orifice of Fruchaud was exposed properly and all the potential hernia sites (direct/ indirect) and femoral were dissected. A 15 × 12 cm polypropylene mesh was introduced with 10-mm trocar and placed after unrolling it in the preperitoneal space. All 3 potential hernia sites were covered. The mesh was placed from pubic symphysis (overlapping 2 cm to the opposite side) to the anterior superior iliac spine laterally. No mesh fixation was done. The CO_2_ desufflation was done under vision. The port sites were assessed for hemostasis. The facial defects were closed after complete emptying of CO_2_ from the extraperitoneal space. The skin incision was closed with absorbable sutures.

All the open Lichtenstein mesh repair procedures were performed as standard tension-free mesh repair. A polypropylene mesh of 12 × 8 cm was fixed with 2-0 Prolene. All the cord structures and ilioinguinal nerve was dissected cautiously. Hemostasis was achieved and closure was done with vicryl 2-0. Skin sutures were taken with vicryl 3-0 subcuticular sutures.

A single shot of ceftriaxone was given preoperatively. Postoperatively paracetamol 500 mg 8 hourly was administered intravenously; if required diclofenac sodium 50 mg was given 12 hourly for the first 24 hours. Oral diclofenac sodium 50 mg was prescribed after 24 hours and injectable analgesic was administered only if the patient had severe pain.

The postoperative pain scoring was done (12 hours postoperatively) by numerical pain score ranging from 0 to 10 (0: no pain, 1–3: mild pain, 4–7: moderate pain, 8–10: severe pain). The length of hospital stay was recorded. Patients were followed-up on day 7, 1 month, and 6 months postoperatively. They were evaluated for pain, wound infection, resumption of normal activities, numbness/burning at the operative site, chronic pain, or recurrence of the hernia.

### 2.2. Statistical analysis

Continuous data are presented in the form of mean ± standard deviation and compared using an independent *t* test, whereas categorical data are presented in frequency (%) and compared using the chi-square test. Statistical software named “MedCal-12.2.1” was used for analysis. Significance is set at 5% in this study. All *P* values <.05 were considered statistically significant in this study.

## 3. Results

As shown in Table 1, 88 patients were included in the TEP group A with a mean age of 47.4 ± 14.1 (range 18–75 years), and 86 patients were included in the Lichtenstein repair group B with a mean age of 50.05 ± 13.73 (range 18–80 years). The male to female ratio was 84:2 and 85:3 in TEP and Lichtenstein groups, respectively.

**Table 1:** Demographic details and types of hernias.

Variables	TEP, group A (N=88)	OLR, group B (N=86)	*P* value
Age	47.4 ± 14.1	50.05 ± 13.73	.211
Gender	M/F = 84/2	M/F = 85/3	.979
Right side	54 (61.4%)	49 (56.9%)	.664
Left side	34 (38.6%)	37 (43.02%)	
Indirect hernia	71 (80.6%)	17 (19.7%)	<.001
Direct hernia	14 (15.9%)	67 (77.9%)	
Pantaloon hernia	3 (3.4%)	2 (2.3%)	

M/F = male/female, OLR = Open Lichtenstein tension-free mesh repair, TEP = total extraperitoneal.

There were no statistical differences in the laterality of the hernia in both groups. Indirect hernias were 71 (80.6%) and 17 (19.7%), whereas direct hernias were 14 (15.9%) and 67 (77.9%), respectively, in TEP and Lichtenstein groups. Pantaloon hernias were 3 (3.4%) and 2 (2.3%) in TEP and Lichtenstein groups, respectively.

The occupations and predisposing factors are enlisted in Table 2.

**Table 2:** Occupation and predisposing factors in both groups.

Occupation factors	TEP, group A (N = 88), n (%)	OLR, group B (N = 86), n (%)
None	24 (27.2)	18 (20.9)
Sitting/desk job	18 (20.45)	22 (25.5)
Light work	32 (36.3)	28 (32.5)
Heavy manual work	14 (15.9)	18 (20.9)
**Predisposing factors**		
None	39 (44.3)	34 (39.5)
Long standing work	18 (20.4)	21 (24.4)
Heavy weight lifting	13 (14.7)	11 (12.7)
Constipation	6 (6.8)	5 (5.8)
Difficulty in micturition	8 (9)	9 (10.4)
Chronic cough/COPD	4 (4.5)	6 (6.9)

COPD = chronic obstructive pulmonary disease, OLR = Open Lichtenstein tension-free mesh repair, TEP = total extraperitoneal.

All patients for TEP group A were operated under general anesthesia. In LMR group B, 73 patients (84.8%) were operated under spinal anesthesia, 10 patients (11.6%) were operated under epidural anesthesia, and 3 patients (3.4%) were operated under general anesthesia due to difficult spinal anesthesia.

Postoperative pain at various time intervals are shown in Table 3. On postoperative day 1, percentage of mild pain reported was more, that is, 60.2%, in laparoscopic TEP group A as compared to 13.9% in Lichtenstein repair group B. This difference was statistically significant (*P* < .001).

**Table 3:** Postoperative pain at different time intervals in both groups.

	Day 1			Day 7			1 mo			6 mo		
Numerical pain scale	TEP (N = 88), n (%)	OLR (N = 86), n (%)	*P* value	TEP (N = 88), n (%)	OLR (N = 86), n (%)	*P* value	TEP (N = 88), n (%)	OLR (N = 86), n (%)	*P* value	TEP (N = 88), n (%)	OLR (N = 86), n (%)	*P* value
No pain: 0	0	0	<.001	3 (3.4)	0	<.001	46 (52.2)	9 (10.4)	<.001	71 (80.6)	62(72)	.409
Mild: 1–3	53 (60.2)	12 (13.9)		69 (78.4)	48 (55.8)		39 (44.3)	72 (83.7)		15 (17)	21 (24.4)	
Moderate: 4–7	26 (29.5)	61 (70.9)		16 (18.1)	26 (30.2)		3 (3.4)	5 (5.8)		2 (2.2)	3 (3.4)	
Severe: 8–10	7 (7.9)	13 (15.1)		1 (1.13)	12 (13.9)		0	0		0	0	

OLR = Open Lichtenstein tension-free mesh repair, TEP = total extraperitoneal.

On postoperative day 1, moderate pain was reported more in Lichtenstein repair group B (70.9%) compared to laparoscopic TEP group A (29.5%).

Severe pain was reported in 7.9% and 15.1% patients in laparoscopic TEP group A and the Lichtenstein repair group B, respectively, which was statistically significant (*P* < .001).

Also, at day 7, the difference in the moderate and severe pain reported in Lichtenstein repair group B was statistically significant as compared to TEP group A (*P* < .001).

The mean duration of time of operation for laparoscopic TEP group A was 84.6 ± 32.2 minutes and for the Lichtenstein group B was 59.2 ± 14.8 minutes, which showed a statistically significant difference (*P* < .001). Tables 4 and 5 show postoperative complications and postoperative outcomes between the 2 groups, respectively.

**Table 4:** Postoperative complications in both groups.

Postoperative complications	TEP, group A (N = 88), n (%)	OLR, group B (N = 86), n (%)
Hematoma	4 (4.5)	2 (2.3)
Seroma	7 (7.9)	3 (3.4)
Scrotal swelling/testicular pain	3 (3.4 )	3 (3.4)
Spermatic cord edema	2 (2.2)	8 (9.3)
Wound infection	2 (2.2)	4 (4.6)
Urinary complaints	6 (6.8)	3 (3.4)

OLR = Open Lichtenstein tension-free mesh repair, TEP = total extraperitoneal.

**Table 5:** Postoperative outcomes in both groups.

Postoperative outcomes	TEP, group A (%) (N = 88), n (%)	OLR, group B (N = 86), n (%)	*P* value
Hospital stay (d), mean ± SD	2.68 ± 1.52	3.86 ± 6.16	.083
Return to normal activities (d), mean ± SD	13.6 ± 6.8	19.8 ± 4.6	<.001
Chronic groin pain	3 (3.4)	19 (22.09)	<.001
Numbness or burning of inguinoscrotal region	4 (4.5)	13 (16.2)	.036
Recurrence	2 (2.2)	1 (1.6)	.984

OLR = Open Lichtenstein tension-free mesh repair, SD = standard deviation, TEP = total extraperitoneal.

The length of hospital stay was less in laparoscopic TEP group A (2.68 ± 1.52 days) as compared to that in Lichtenstein repair group B (3.86 ± 6.16 days; *P* = .083).

Return to normal activities was earlier in TEP group A 13.6 ± 6.8 days in comparison with 19.8 ± 4.6 days in Lichtenstein group B, with a significant *P* value (*P* < .001).

The postoperative complications like chronic groin pain were observed in 19 patients (22.09%), whereas numbness or burning of the inguinoscrotal region was seen in 13 patients (16.2%) in Lichtenstein repair group B, which were seen in less number of patients, 3 (3.4%) and 4 (4.5%) patients, respectively, in TEP group A. The difference in the recurrences rates was not statistically significant (*P* = .984) in both groups.

## 4. Discussion

Tension-free hernia mesh repair was introduced by Lichtenstein and Shulman.^[6]^ Lichtenstein tension-free hernia repair has become the gold standard and the most suitable technique for open hernia repair with promising results and low recurrence rates.^[4,7]^ After the evolvement of laparoscopy in hernia repair claiming similar results, several studies were performed to compare it with open repair techniques.^[8–10]^ There were controversial results of laparoscopic hernia techniques.^[11]^

Some studies in the literature have stated that the laparoscopic TEP technique has been deliberated as a procedure of choice for laparoscopic hernia repair as the peritoneal cavity is not breached.^[12]^ In this study, we have compared the 2 most acceptable procedures for inguinal hernia repair between the 2 groups with similar demographic features.

One hundred seventy-four patients were included in the study. Eighty-eight patients were operated on by laparoscopic TEP and 86 by open Lichtenstein tension-free mesh repair. The mean operative time for Lichtenstein mesh repair was 59.2 ± 14.8 minutes standard deviation and for TEP it was 84.6 ± 32.2 minutes in our study. There are studies in the literature reporting the longer operating time for laparoscopic hernia repair as compared to open repair.^[13,14]^

Some studies have reported a similar operative time in open and laparoscopic hernia repair.^[15]^ The learning curve of laparoscopic hernia surgery is longer as reported in the various literature.^[16–19]^ Table 6 shows comparison of operative time in various studies as described in the literature.^[20–24]^

**Table 6:** Various studies showing the operative time.

Studies	Operative time in laparoscopic hernia repair (min)	Operative time in open hernia repair (min)
Wright et al (1996)^22^	58	45
Picchio et al (1999)^21^	49.6	33.9
MRC trial group (1999)^20^	58.4	43.3
Anderberg et al (2003)^23^	50	45
Kouhia et al (2009)^24^	69 ± 40	58 ± 16
Present study	84.6 ± 32.2	59.2 ± 14.8

In the laparoscopic TEP group, the main intraoperative complication was the accidental creation of pneumoperitoneum, which occurred in 13 cases (14.7%), later managed by insertion of Veress needle/5-mm port at Palmer’s point to decompress the pneumoperitoneum. There was no conversion to open surgery.

A higher level of pain has been reported in the previous studies after open hernia repair procedures like Lichtenstein hernioplasty as compared to laparoscopic hernia repair.^[25,26]^

In this study, on postoperative day 1, severe pain was reported in 7.9% and 15.1% patients in laparoscopic TEP group A and Lichtenstein repair group B, respectively, which was statistically significant (*P* < .001). Moderate pain was reported more in the Lichtenstein repair group B (70.9%) as compared to that in laparoscopic TEP group A (29.5%).

The mean postoperative pain in laparoscopic hernia repair has been reported to be lower as compared to open procedures.^[27]^ Other studies state that at postoperative day 7, the pain level in laparoscopic hernia repair is less as compared to open hernia repair procedure.^[28–30]^

At postoperative week 1, the pain levels were significantly less in the laparoscopic TEP group (moderate pain in 18.1%) than in the Lichtenstein group (moderate pain in 30.2%), which is a statistically significant difference (*P* < .001). Severe pain was reported by 13.9% in the Lichtenstein group as compared to 1.13% in the laparoscopic TEP group.

Postoperative follow-up at 1 month and 6 months showed more percentage of mild and moderate pain in the Lichtenstein group as compared to the laparoscopic TEP group. No severe pain was reported in any of the groups at a 1-month and 6-month postoperative period. This is well in correlation with the findings reported by Courtney et al.^[31]^

Thus, patients undergoing the laparoscopic procedure, in this study as well as other studies, have reported lower rates of postoperative pain compared to the open techniques.^[32,33]^

In this study, the occurrence of postoperative complications like hematoma, wound infection, scrotal swelling, and testicular pain were not statistically significant in both groups. Seroma formation was more in the laparoscopic TEP group (7.9%) as compared to the Lichtenstein group (3.4%). Spermatic cord edema was more in the Lichtenstein group (9.3%) as compared to the laparoscopic TEP group (2.2%). This was in contrast to the study reported by Feliu et al,^[34]^ the postoperative complications were more frequent in the open hernia repair group (23.9%) than in the TEP group (13.9%).

Chronic pain was seen in a significantly higher number of patients in the Lichtenstein group vs the laparoscopic TEP group (22.09% vs 3.4%). This was in accordance with the meta-analysis reported by Bobo et al.^[35]^

Literature shows debatable reports in view of chronic pain. Meta-analyses by Karthikesalingam et al^[36]^ and Dhankhar et al^[37]^ reported no significant difference in chronic pain in laparoscopic and open mesh repair patients. Other several studies have reported higher incidences of chronic groin pain in open repair patients as compared to laparoscopic repair patients.^[26,38–41]^

Persistent chronic groin pain affects the quality of life of the patients. The possible reason for the chronic groin pain postoperative in open hernia mesh repair is unclear. It can be due to nerve injury/nerve entrapment, quality of mesh used in the repair, or maybe due to improper positioning of mesh in the inguinal canal.^[42,43]^ However, we need to consider that many patients may report groin discomfort as chronic groin pain.

The length of hospital stay in the postoperative period was significantly less in the laparoscopic TEP group vs Lichtenstein group (2.68 ± 1.52 vs 3.86 ± 6.16), which was in accordance with the study by Lal et al.^[32]^ However, the length of hospital stay was similar in open repair group vs Lichtenstein group in the long-term follow up study by Eker et al.^[33]^

The duration of time taken to resume normal activities was significantly lower in the laparoscopic group (13.6 ± 6.8) vs (19.8 ± 4.6) in the Lichtenstein group, which was statistically significant (*P* < .001). Similar results were seen in the studies reported by O’Brien et al^[32]^ and other studies in the literature.^[36]^ So the patients who underwent laparoscopic TEP could join their work earlier than the Lichtenstein group.

To evaluate the better surgical technique for inguinal hernia repair, the recurrence rate was the main outcome factor observed for many years. Lichtenstein’s tension-free mesh repair technique is considered the gold standard for inguinal hernia repair as it reduced the recurrence rate to <1%.^[44,45]^

There are controversial reports in view of recurrences seen in open and laparoscopic hernia repair. In this study, 2 recurrences (2.2%) were seen in laparoscopic TEP group and 1 recurrence (1.6%) in the Lichtenstein group. This was in accordance with the study by Bariş et al,^[46]^ which showed recurrence in 3.4% of cases in the TEP group and 5.2% of cases in the open Lichtenstein group. Various studies, meta-analyses, and trial sequential analyses report no difference in recurrence rates between laparoscopic and open inguinal hernia repair techniques.^[36,47,48]^ Champault et al^[28]^ showed (2% vs 6%) recurrences rates in their study in laparoscopy repair vs open repair.

TEP and Lichtenstein for inguinal hernia repair have been evaluated by various clinical trials showing no conclusive evidence of differences in terms of outcomes like chronic pain, complications, and recurrence. In primary unilateral inguinal hernia and in bilateral cases, the laparo-endoscopic approach (TEP, transabdominal preperitoneal) is the first choice, provided the surgeon has sufficient expertise as reported in their report as a part of International Guidelines by various hernia societies.^[49]^

There are limitations in this study, like smaller sample size and shorter follow-up duration. The recurrent and bilateral hernias could not be evaluated because of exclusion from the study to evaluate the similar groups without affecting the results. Another limitation was the difference in the type of anesthesia in both the groups because open hernia repair was acceptably done under spinal anesthesia and laparoscopic repair was done under general anesthesia.

## 5. Conclusion

Laparoscopic TEP repair can be offered safely with comparable results and acceptable outcomes like less postoperative pain, early resumption of work, and better cosmetic results in comparison with open mesh repair. Although the open Lichtenstein hernia repair is largely accepted by many surgeons as easy to perform and cost-effective with the lowest recurrence rates but having long-term complications like chronic groin pain and late resumption of work in the patients.

Laparoscopic TEP can be a better procedure of choice in bilateral and recurrent inguinal hernias which needs to be evaluated with long-term follow-up studies.

At present Laparoscopic TEP hernia repair can be a better alternative to open procedure and feasible to be done with acceptable results if performed by experienced surgeons.

## Acknowledgments

Mr Vikas Doshi (Bio-Statistician) has helped in the statistical work for this study.

## Author contributions

Mohammed Yunus Shah—conceived and designed the analysis, collected the data, wrote the article, corresponding author.

Pratik Raut—contributed data and analysis tool.

T. R. V. Wilkinson—performed the analysis, conceived, and designed the analysis.

Vijay Agrawal—collected the data.
